# Riverbed Substrate Requirements for Natural Reproduction of *Gymnocypris przewalskii*

**DOI:** 10.3390/ani11113246

**Published:** 2021-11-13

**Authors:** Yanghao Zhou, Junyi Li, Hongfang Qi, Haile Yang, Xuan Ban, Jianxin Yang, Hao Du

**Affiliations:** 1 Key Laboratory of Freshwater Biodiversity Conservation, Ministry of Agriculture and Rural Affairs of China, Yangtze River Fisheries Research Institute, Chinese Academy of Fishery Sciences, Wuhan 430223, China; zhouyanghao0424@126.com (Y.Z.); LJY_IUI@163.com (J.L.); haileyang18@yfi.ac.cn (H.Y.); 2 College of Fisheries, Huazhong Agricultural University, Wuhan 430037, China; 3 Qinghai Key Laboratory of Breeding and Protection of Naked Carp, Qinghai Lake Naked Carp Rescue Center, Xining 810016, China; qhf1970@163.com (H.Q.); yjxh10@126.com (J.Y.); 4 Hubei Key Laboratory of Environmental and Disaster Monitoring and Assessment, Innovation Academy of Precision Measurement Science and Technology, Chinese Academy of Sciences, Wuhan 430077, China; banxuan@apm.ac.cn

**Keywords:** *Gymnocypris przewalskii*, riverbed substrate, natural reproduction, artificial simulation

## Abstract

**Simple Summary:**

Riverbed substrate plays a vital role in the growth, reproduction, predation of fish as an important aspect of a river ecosystem. This research represents the first systematic examination of the riverbed substrate requirements for the natural reproduction of *Gymnocypris przewalskii* through artificial simulation and spawning ground substrate transformation experiments. Results showed that pebble was an indispensable factor for the reproduction of *Gymnocypris przewalskii* and the increase of pebble had an obvious promoting effect on the reproduction. The findings of this study could provide guidance for the wild population recovery, artificial reproduction, and restoration of natural spawning grounds of this fish species.

**Abstract:**

*Gymnocypris przewalskii* (i.e., Qinghai Lake naked carp) is a migratory fish species that lives in highland brackish water. It is important to understand the abiotic environment required by this fish to reproduce naturally so that its habitat can be protected and the wild population can be conserved. Here, artificial simulation and spawning ground substrate transformation experiments were conducted to examine the riverbed substrate requirements for *G. przewalskii* to naturally reproduce. Using various techniques (in vitro markers, videography, and Ethovision XT behavior tracking), this study systematically investigated the riverbed substrate preferences of *G. przewalskii* as well as the characteristics and effectiveness of natural reproduction induced by pebble riverbed substrate. The findings can be summarized as follows: (1) the habitat preferences of *G. przewalskii* differed significantly between various riverbed substrate, with pebble substrate being preferred during natural reproduction, and sand substrate being preferred pre- and post-spawning, and (2) the natural reproduction of *G. przewalskii* was heavily reliant on pebble riverbed substrate. Specifically, pebble substrate significantly improved spawn quantity and fertilization rate. These findings provide scientific evidence for the improvement and restoration of *G. przewalskii* spawning grounds, and insights regarding the artificial bionic reproduction of *G. przewalskii*.

## 1. Introduction

As an important part of a river ecosystem [1], riverbed substrate play an important role in the growth, reproduction, predation, and behavior of fish [2,3,4,5]. For fish spawning within bottom sediments, the grain diameter, composition, and spatial structure of riverbed substrate can affect the occurrence and effectiveness of their natural reproduction [6,7]. For example, artificial riverbed substrate can be created to induce the natural spawning in sturgeon (such as *Acipenser fulvescens* and *Acipenser ruthenus*) [8,9]. Evidently, riverbed substrate plays an important role in the natural reproduction cycles of fish spawning within bottom sediments. Therefore, understanding the riverbed substrate requirements for natural reproduction is of great significance to conservation and habitat restoration for threatened fish species.

*Gymnocypris przewalskii* is a fish within the family Cyprinidae and the order Cypriniformes; it is the only species of commercial fish living in Qinghai Lake and is also rated as an endangered species in the *China Species Red List*. Qinghai Lake is located in the Qinghai-Tibet Plateau of China with an average altitude of 3200 m, which is the Chinese largest highland saltwater lake. The lake’s extreme hydrological and climatic conditions cause *G. przewalskii* to grow very slowly [10]. From May to July every year, sexually mature *G. przewalskii* individuals engage in anadromous migration and reproduction on coarse bottoms [11], making *G. przewalskii* a typical anadromous fish species [12]. From the early 1960s to late 1990s, the available stock of *G. przewalskii* plummeted from 690 million to 25 million due to anthropogenic activities (e.g., overexploitation of resources, overfishing, and habitat destruction). It was not until the late 20th century that the conservation of *G. przewalskii* began to be given due attention [13]. Currently, however, only five rivers are available for the spawning migration of *G. przewalskii*; the suitable spawning ground area for *G. przewalskii* has decreased drastically under the influence of damming and floodplain transformation [14]. Based on the ecological habit characteristics of the bottom spawning of *G. przewalskii*, this study aimed to determine the riverbed substrate required for them to naturally reproduce. This will provide scientific evidence for the wild population recovery and spawning ground restoration of *G. przewalskii*.

## 2. Materials and Methods

### 2.1. Selection of Experimental Fish

Fishing with trawl was carried out to obtain the parental fish of *G. przewalskii* in the Quanji and Shaliu Rivers. Length, width, and mesh of the trawl were 3 cm, 16 m, and 2 m, respectively. Before the experiments, parental fish with well-developed gonads (at least Stage IV) and obvious secondary sex characteristics were selected through ultrasound observations and transferred to the experimental site by temporary incubator without mortality. With a sex ratio of 1:1 in each case, 36 and 72 wild-caught parental fishes were used in the riverbed substrate preference and riverbed substrate inducement experiments, respectively. In these two experiments, the body lengths of male parental fish were 181.5 ± 30.5 and 187.5 ± 36.5 mm, respectively (mean ± standard deviation); the body weights of male parental fish were 68.6 ± 34.5 and 68.4 ± 34.3 g, respectively. The corresponding lengths and weights of female parental fish were 203.5 ± 52.5 and 198.5 ± 34.5 mm, respectively, and 103.6 ± 65.1 and 105.9 ± 63.2 g, respectively. Table 1 describes the detailed biological characteristics of the parental fish.

### 2.2. In Situ Observation of Natural Spawning Ground

**Figure 1:**
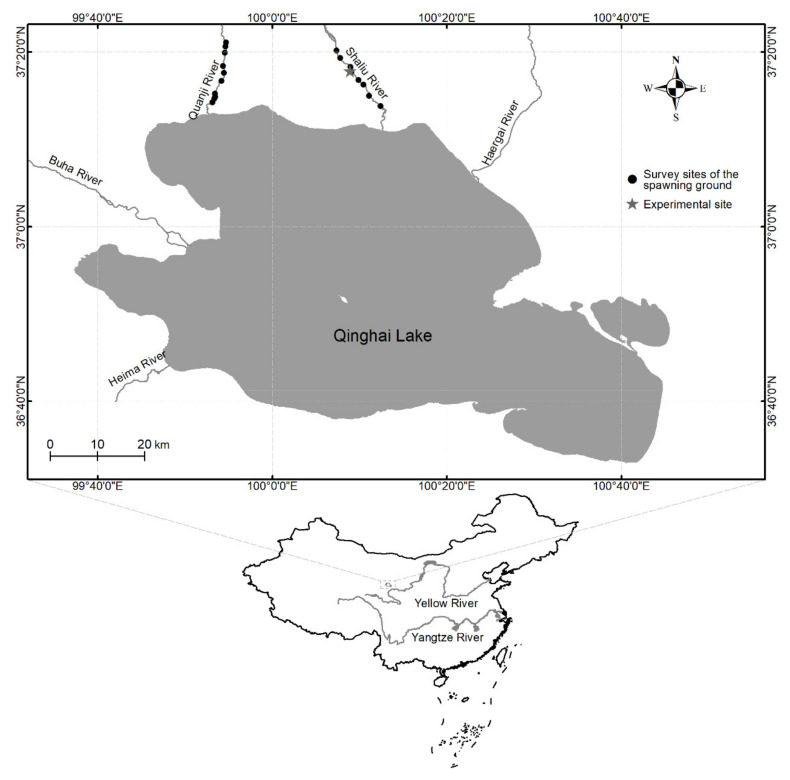
In situ observations and measurements sites of the spawning grounds in Shaliu and Quanji Rivers.

Before this study was conducted, in situ observations and measurements were conducted to determine the flow velocity, photoperiod, water depth, temperature, and riverbed substrate of the spawning grounds (See Figure 1) of *G. przewalskii* during their natural reproduction period. This was done to aid the creation of an artificial spawning environment for *G. przewalskii*. Table 2 describes the environmental parameters of the natural spawning grounds of *G. przewalskii*.

### 2.3. Riverbed Substrate Reference Experiment 

**Figure 2:**
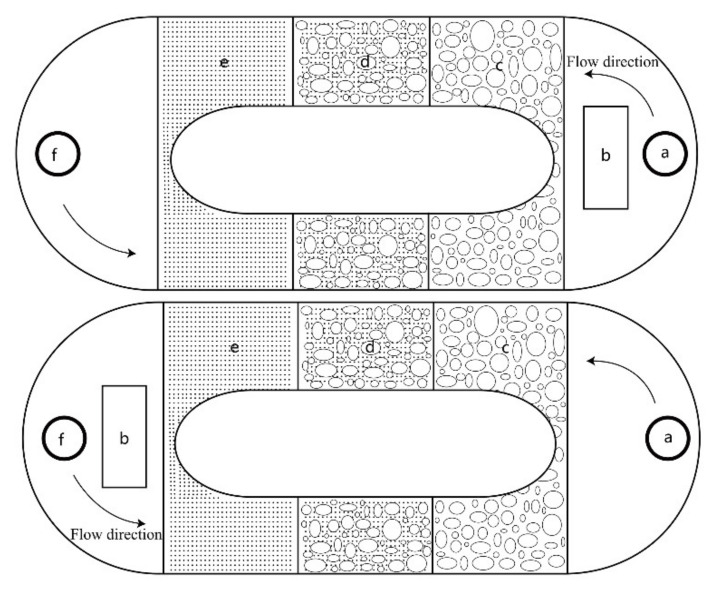
Diagrammatic sketch of the riverbed substrate reference experiment. (**a**,**b**,**f**) The water inlet, water pump, and outlet, respectively; (**c**–**e**) three types of substrate: pebbles, pebbles blended with sand, and pure sand, respectively.

The substrate preference experiment for *G. przewalskii* was conducted during its natural reproduction period in a polyethylene circular pond with a diameter of 3.3 m and a water depth and velocity of 0.2 m and 0.2–0.4 m/s. Three types of substrate (pebbles with a grain diameter of 20–180 mm, pebbles blended with sand, and pure sand with a grain diameter of less than 1 mm), each with an equal area (1.1 m × 0.5 m), were laid and distributed in the form of upper and lower mirror images in this circular pond. In the pebble area, ratio of pebble with different grain diameter (20–50 mm, 50–100 mm, and 100–180 mm) was 2:3:5, respectively, and in the area of pebble blended with sand, the ratio of pebble to sand was 7:3. Partition meshes were set up at two ends of the circular pond to separate the experimental observation area from the environment creation devices (a water pump, water inlet, and water outlet). In order to avoid the influence of outlet velocity on selection preference, two parallel groups with pebble area or sand area as the initial flow generation point were used in the experiment (See Figure 2). There were 3 male and 3 female parent fish in each group, and the experimental period was 24 h. The water for the experiment was acquired from the Shaliu River; it was filtered and aerated before use. The pebbles and sand used in the experiment were acquired from the Shaliu River. The experiment was repeated three times.

### 2.4. Riverbed Substrate Inducement Experiment

**Figure 3:**
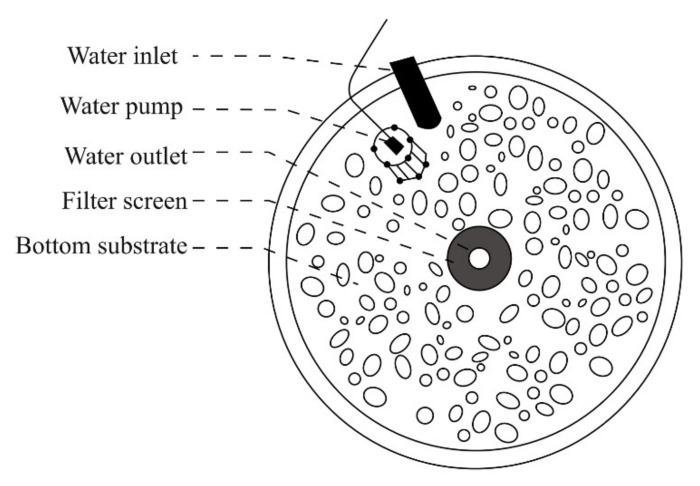
Diagrammatic sketch of the riverbed substrate inducement experiment.

Based on the results of the substrate preference experiment (Section 2.3), an experiment was conducted in a polyethylene circular pond with a diameter of 1.5 m and a water depth of 0.2 m to further verify whether pebble substrate could induce the natural reproduction of *G. przewalskii* (See Figure 3). Three parallel tests were conducted, so this experiment comprised the following experimental groups: a blank group, a sand group (grain diameter: <1 mm), and a mud group. Three female and three male parental fish were selected for each experimental group; the experimental period was 24 h. Each test was repeated four times, with all tests being conducted simultaneously. In the experimental groups without any spawning, pebble substrate was added; experimental results were observed for 12 h. The riverbed substrate used in the experiment were all acquired from the habitats of *G. przewalskii*.

### 2.5. Field Riverbed Substrate Transformation Experiment 

**Figure 4:**
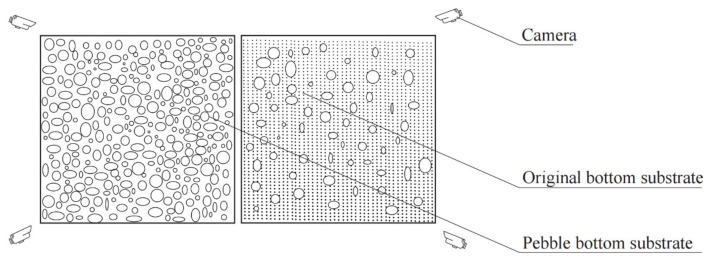
The field riverbed substrate transformation experiment.

To verify the habitat and reproduction preferences of *G. przewalskii* in natural spawning grounds with pebble substrate, experimental fish comprising parental fish populations acquired from natural spawning grounds were used; the experimental period was 24 h. The habitat preferences of *G. przewalskii* were assessed by videoing the areas where the fish schools stayed. Fish roe was obtained by taking out the whole riverbed substrate from the experimental area with a shovel, and the diurnal spawning quantity and fertilization rate of *G. przewalskii* in each area were then assessed by counting the number of fish roe. The riverbed substrate was then artificially transformed based on the natural spawning grounds of *G. przewalskii*. Specifically, area A was an artificially transformed natural spawning ground by artificially adding more pebbles than that of area B, whereas for area B, the original riverbed substrate (pebbles with sand) of the natural spawning grounds was retained. Both areas were 1.5 m × 1.5 m in size (See Figure 4). In addition, purse seine was used to fence the experimental areas so that a controlled area could be provided during the whole experiment.

### 2.6. Parental Fish Marking and Videography 

**Figure 5:**
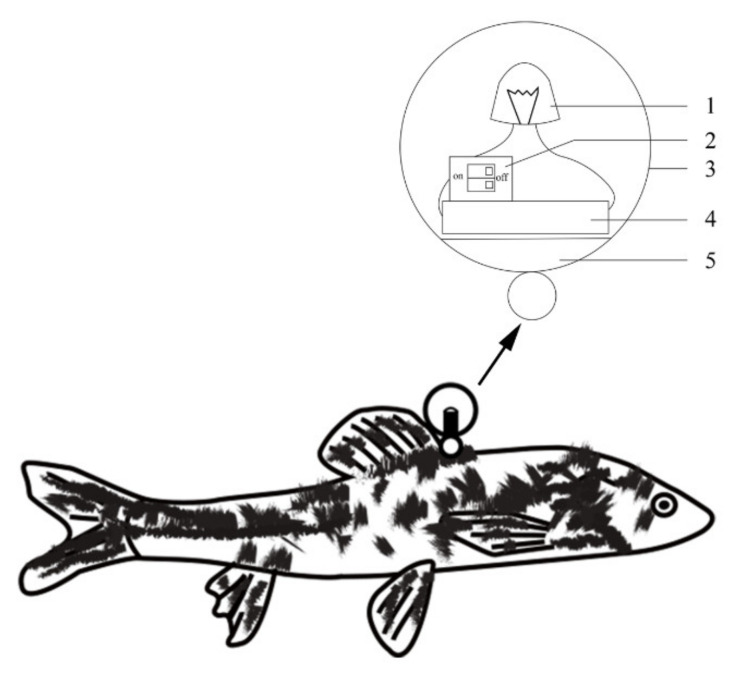
Composition and fixed position of the optical waterproof marker. Panels (**1**–**5**) represent the light source, switch, shell, power, and cover body, respectively.

In the experiments specified in Section 2.3 and Section 2.4, in vivo optical waterproof markers were added to the experimental parental fish and a video camera was fixed above each experimental pond for continuous video recording. Each in vivo optical waterproof marker comprised five parts: a light source, switch, shell, cover body, and power supply; the whole set weighed 5 g, with a diameter of 30 mm (See Figure 5).

### 2.7. Data Processing and Analysis 

In each experimental group, the temporal sampling rate of video image analysis was 10 min·h^−1^, and the total durations of video analysis were 240 and 360 min for the 24 and 36 h experimental groups, respectively. The Ethovision XT14 (Noldus, Wageningen, Netherlands) was used to process and analyze video image data, and to determine the movement trajectories of individual parental fish and their residence times in the different riverbed substrate areas. Upon completion of these experiments, the spawning quantity and fertilization rate of each experimental group were calculated. Difference comparisons and analyses were conducted using the one-way analysis of variance (ANOVA) and Duncan functions in SPSS 22.0.

## 3. Results

### 3.1. Habitat Preferences of Parent Fish Regarding Riverbed Substrate

**Figure 6:**
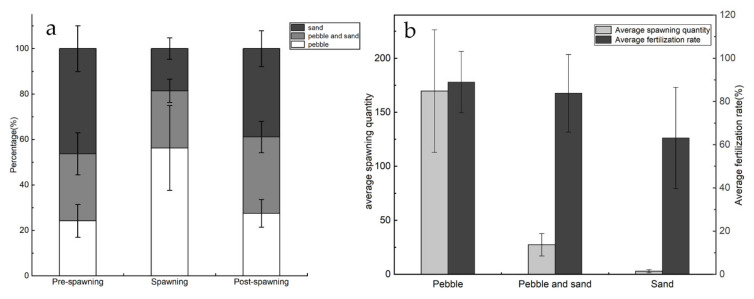
The proportional residence times (**a**) and average spawning quantity and fertilization rate (**b**) of *G. przewalskii* in the different riverbed substrate and time.

At the pre-spawning, spawning, and post-spawning stages, the residence times of *G. przewalskii* parental fish varied significantly in different riverbed substrate environments (*p* < 0.05): (1) during the pre-spawning stage, the proportional residence times in the different riverbed substrate environments were 24.2 ± 14.1% (pebble substrate), 29.5 ± 7.1% (pebble and sand substrate), and 46.3 ± 8.4% (pure sand substrate). (2) During the spawning stage, the proportional residence times in the different riverbed substrate environments were 56.3 ± 17.6% (pebble substrate), 25.1 ± 6.1% (pebble and sand substrate), and 18.6 ± 18.8% (pure sand substrate). (3) During the post-spawning stage, the proportional residence times in the different riverbed substrate environments were 27.5 ± 17.6% (pebble substrate), 33.6 ± 6.1% (pebble and sand substrate), and 38.9 ± 18.8% (pure sand substrate) (See Figure 6a). 

Statistical analysis revealed that the average spawning quantity of *G. przewalskii* parental fish were 169.7, 2.9, and 27.4 in the pebble, pebble and sand, and pure sand substrate environments, respectively; the average fertilization rate was 89.01, 83.77, and 63.13%, respectively (See Figure 6b). The spawning effectiveness therefore different significantly between the three substrate environments.

### 3.2. Effectiveness of Reproduction Induced by Addition of Pebble 

The riverbed substrate inducement experiment (Section 2.4) revealed that *G. przewalskii* did not spawn on blank or mud substrate but reproduced naturally at a very small scale on the pure sand substrate group (only one group). 

**Figure 7:**
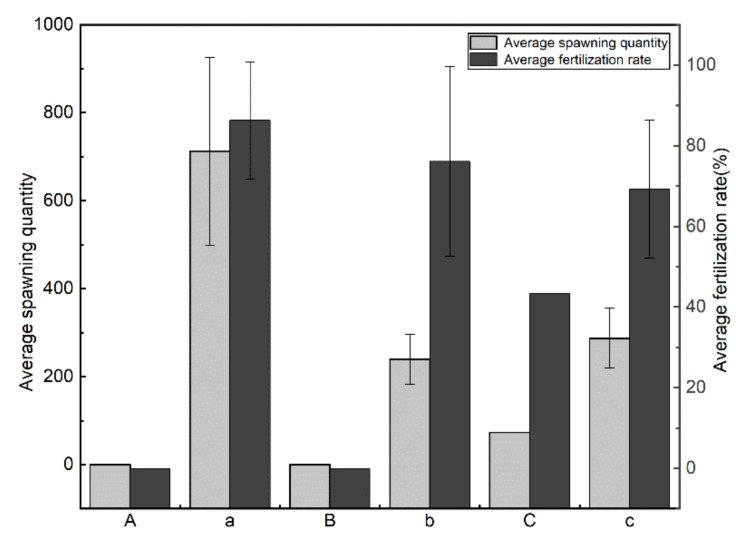
Average spawning quantity and fertilization rate of each group. (**A**–**C**) The blank group, sand group, and mud group, respectively; (**a**–**c**) the above groups after adding pebbles.

After pebbles were artificially added to all experimental groups, natural reproduction occurred in all experimental groups, except for one experimental group with a mud substrate. After pebbles were added in the experimental group with pure sand substrate, the spawning quantity increased from 73 to 287 and the fertilization rate increased from 43.25% to 69.17%. This indicates that there were significant changes in spawning scale and effectiveness. After pebble substrate were added to all experimental groups, the average spawning quantity (712) and fertilization rate (86.26%) of the blank group were the highest (See Figure 7). This showed that *G. przewalskii* could spawn more effectively on pure pebble substrate.

### 3.3. Effectiveness of Reproduction from Transformation of Natural Spawning Ground 

**Figure 8:**
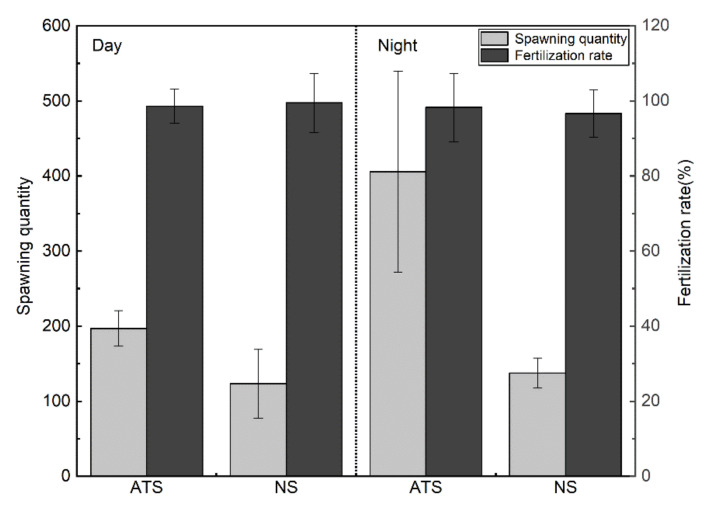
Spawning quantity and fertilization rate of *G. przewalskii in* artificially transformed substrate and natural riverbed substrate in the day and night. ATS represented the artificially transformed substrate, and NS represented the natural substrate.

Continuous 24 h observations revealed the following results: (1) during the day, the spawning quantity of *G. przewalskii* was 197 and 123 on the artificially transformed pebble and natural riverbed substrate, respectively. The fertilization rate of *G. przewalskii* for these two substrates was 98.6 and 99.5%, respectively. (2) At night, the spawning quantity of *G. przewalskii* on the artificially transformed pebble and natural riverbed substrate were 406 and 138, respectively; the corresponding fertilization rate was 98.3 and 96.7%, respectively (See Figure 8). Thus, the spawning quantity of *G. przewalskii* in the artificially transformed spawning ground were significantly larger than those in the natural spawning ground, whereas the fertilization rate of *G. przewalskii* did not differ significantly between the two. Moreover, video observations revealed that *G. przewalskii* reproduced more actively and spawned more eggs at night than they did during the day, and that they preferred to spawn on pure pebble substrate.

## 4. Discussion

Compared with the complex, varied, and uncontrollable environments of natural spawning grounds, an artificially regulated biotic environment is relatively stable. However, certain factors (e.g., spatial limitations, unnatural environmental conditions, and the capture and transportation of tested fish) in an artificially regulated biotic environment can impact fish behavior. In this study, riverbed conditions were regulated by repeated simulation via substrate inducement in an artificially regulated environment. This was done during the early stages of the experiments to achieve the natural and stable mating and spawning of *G. przewalskii* and achieve reliable and consistent results. However, other environmental factors (e.g., water flow and water temperature) may also affect the natural reproduction behavior of *G. przewalskii*; the specific impacts of other environmental factors should therefore be further examined.

Here, pebble substrate was artificially added to successfully induce the spontaneous reproduction of *G. przewalskii* in an artificially simulated spawning environment. The pebble substrate was then regulated to significantly increase the inducement rate, spawning quantity, and fertilization rate. This indicates that pebbles are an indispensable factor for the reproduction of *G. przewalskii*. However, the mechanistic role that pebbles play in this reproductive process remains unclear. Many studies have argued that riverbed substrate and landforms determine the characteristics of the flow field in a spawning ground. Thus, it has been suggested that the selection of pebble-related habitats for reproduction is probably driven by other variables (e.g., hydrodynamics), rather than by the specific physical characteristics of pebbles or their associated substrate [15]. Water flow accelerates around riverbed pebbles, thus eroding the materials around the pebbles and removing sediments from the riverbed over time [16]. As exemplified by sturgeon, the presence of a clean riverbed facilitates the formation of relevant flow microhabitats and clean riverbed substrate, which are required for embryonic development [8,17,18,19]. Moreover, the aquatic organisms within a marine ecosystem are prone to be attracted by the “zone of wake” formed by coral reefs; this principle is similar to the role that pebbles play in a river ecosystem [20]. The flow of river water partly prevents the siltation of riverbed substrate. It also increases the oxygen content of the riverbed bottom, thus facilitating the hatching and embryonic development of fish roe [21,22,23]. Therefore, the micro-flow environment created by the pebbles may be a key factor that induces gonad development and natural reproduction in *G. przewalskii*; this has been widely corroborated for trout [15,21,24]. Subsequent studies should focus on identifying the effects of flow fields with different velocity in riverbed substrate on the reproduction of *G. przewalskii*. This could provide guidance for the wild population recovery, artificial reproduction, and restoration of natural spawning grounds of this fish species.

At present, enhancement and releasing are the major protective measures being employed for *G. przewalskii*. However, an increasing number of studies have shown that there are significant differences in the phenotypic characteristics, behavioral adaptability, and ecological fitness between naturally reproducing wild fish populations and cultured fish populations generated through traditional commercial artificial reproduction patterns (free of natural selection) [25]. Specifically, the field release of artificially reproduced fish individuals may result in extremely low survival rates, obstacles to natural reproduction, potential ecological risks [26,27], and ultimately poor enhancement and release results [28,29,30,31]. To protect endangered fish species in the future, it is therefore necessary to develop reproduction patterns with the aim of improving habitat fitness and maintaining biological instinct of species [32]. Compared with the successful restoration of natural spawning of *Acipenser fulvescens* induced by artificial reefs placement in the river [8], it is more simple and easier to improve the effect of natural reproduction of *G. przewalskii* by increasing the proportion of pebbles in the natural spawning ground. In this study, pebble substrate was added to induce natural reproduction behaviors in wild parental *G. przewalskii*, in an artificially regulated environment. From the perspective of behavioral ecology, this study examined the factors that induce natural reproduction in *G. przewalskii*; the riverbed substrate requirements for natural reproduction were successfully determined. The findings of this study will provide scientific evidence for optimizing artificially simulated reproduction habitats, and for identifying the factors that induce natural reproduction. This study also provides scientific reference for the conservation, development, and utilization of other rare fish species.

## 5. Conclusions

This study represents the first systematic examination of the riverbed substrate requirements for the natural reproduction of *G. przewalskii*, from the perspective of behavioral ecology. Specifically, the influences of riverbed substrate on the natural reproduction behavior and breeding effectiveness of *G. przewalskii* were investigated from three aspects: riverbed substrate preferences in an artificially regulated environment, riverbed substrate inducement, and the transformation of natural spawning grounds. The conclusions of this study can be summarized as follows:

(1) In an artificially regulated environment, *G. przewalskii* significantly preferred pebble riverbed substrate during natural reproduction.

(2) In an artificially controlled environment, *G. przewalskii* did not spawn on riverbed-free, sand, or mud substrate; pebble substrate significantly induced and facilitated natural reproduction.

(3) Increasing the quantity of pebbles in riverbed substrate over natural spawning grounds significantly increased the spawning quantity and fertilization rate.

The results of this study show that *G. przewalskii* significantly prefers to inhabit pebble riverbed substrate during natural reproduction, and that pebbles can both significantly promote the occurrence of natural reproduction and improve its effectiveness.

## Figures and Tables

**Table 1:** Biological parameters of the experimental fish.

Experiment	Number (Ind.)	Sex	Total Length (mm)	Body Length (mm)	Weight (g)
Riverbed substrate preference experiments	18	F	234.5 ± 57.5	203.5 ± 52.5	103.9 ± 65.1
	18	M	220.5 ± 45.5	181.5 ± 30.5	68.6 ± 34.5
Riverbed substrate inducement experiments	36	F	213.5 ± 53.5	198.5 ± 34.5	105.9 ± 63.2
	36	M	195.5 ± 49.5	187.5 ± 36.5	68.4 ± 34.3
Total	54	F	226.0 ± 66.0	210.0 ± 46.0	103.9 ± 65.1
	54	M	205.5 ± 60.5	187.5 ± 36.5	68.4 ± 34.3

**Table 2:** Environmental parameters of the natural spawning grounds of *G. przewalskii*.

Environmental Factor	Value
Velocity	0.2 ± 0.08 m/s
Photoperiod	14:10 (day and light in 24 h)
Water depth	0.2 m
Water temperature	10.3–13 ℃
Ratio of pebble to sand	7:3

## Data Availability

None of the data were deposited in an official repository.
